# Risk of kidney disease following a pregnancy complicated by diabetes: a longitudinal, population-based data-linkage study among Aboriginal women in the Northern Territory, Australia

**DOI:** 10.1007/s00125-023-05868-w

**Published:** 2023-01-18

**Authors:** Matthew J. L. Hare, Louise J. Maple-Brown, Jonathan E. Shaw, Jacqueline A. Boyle, Paul D. Lawton, Elizabeth L. M. Barr, Steven Guthridge, Vanya Webster, Denella Hampton, Gurmeet Singh, Roland F. Dyck, Federica Barzi

**Affiliations:** 1 Menzies School of Health Research, Charles Darwin University, Darwin, NT Australia; 2 Endocrinology Department, Royal Darwin Hospital, Darwin, NT Australia; 3 Clinical Diabetes and Epidemiology, Baker Heart and Diabetes Institute, Melbourne, VIC Australia; 4 Department of Epidemiology and Preventive Medicine, Monash University, Melbourne, VIC Australia; 5 Eastern Health Clinical School, Monash University, Melbourne, VIC Australia; 6 Department of Renal Medicine, Alfred Health, Melbourne, VIC Australia; 7 Central Clinical School, Monash University, Melbourne, VIC Australia; 8 Aboriginal and Torres Strait Islander Advisory Group, Diabetes across the Lifecourse: Northern Australia Partnership, Menzies School of Health Research, Darwin, NT Australia; 9 Central Australian Aboriginal Congress, Alice Springs, NT Australia; 10 Department of Medicine and Canadian Centre for Health and Safety in Agriculture, University of Saskatchewan, Saskatoon, SK Canada; 11 UQ Poche Centre for Indigenous Health, University of Queensland, Brisbane, QLD Australia

**Keywords:** Aboriginal Australians, Chronic kidney disease, Diabetes, gestational, End-stage kidney disease, Indigenous peoples, Oceanic Ancestry Group, Pregnancy complications, Pregnancy in diabetes, Type 2 diabetes

## Abstract

**Aims/hypothesis:**

The aim of this work was to investigate the risk of developing chronic kidney disease (CKD) or end-stage kidney disease (ESKD) following a pregnancy complicated by gestational diabetes mellitus (GDM) or pre-existing diabetes among Aboriginal women in the Northern Territory (NT), Australia.

**Methods:**

We undertook a longitudinal study of linked healthcare datasets. All Aboriginal women who gave birth between 2000 and 2016 were eligible for inclusion. Diabetes status in the index pregnancy was as recorded in the NT Perinatal Data Collection. Outcomes included any stage of CKD and ESKD as defined by ICD-10 coding in the NT Hospital Inpatient Activity dataset between 2000 and 2018. Risk was compared using Cox proportional hazards regression.

**Results:**

Among 10,508 Aboriginal women, the mean age was 23.1 (SD 6.1) years; 731 (7.0%) had GDM and 239 (2.3%) had pre-existing diabetes in pregnancy. Median follow-up was 12.1 years. Compared with women with no diabetes during pregnancy, women with GDM had increased risk of CKD (9.2% vs 2.2%, adjusted HR 5.2 [95% CI 3.9, 7.1]) and ESKD (2.4% vs 0.4%, adjusted HR 10.8 [95% CI 5.6, 20.8]). Among women with pre-existing diabetes in pregnancy, 29.1% developed CKD (adjusted HR 10.9 [95% CI 7.7, 15.4]) and 9.9% developed ESKD (adjusted HR 28.0 [95% CI 13.4, 58.6]).

**Conclusions/interpretation:**

Aboriginal women in the NT with GDM or pre-existing diabetes during pregnancy are at high risk of developing CKD and ESKD. Pregnancy presents an important opportunity to identify kidney disease risk. Strategies to prevent kidney disease and address the social determinants of health are needed.

**Graphical abstract:**

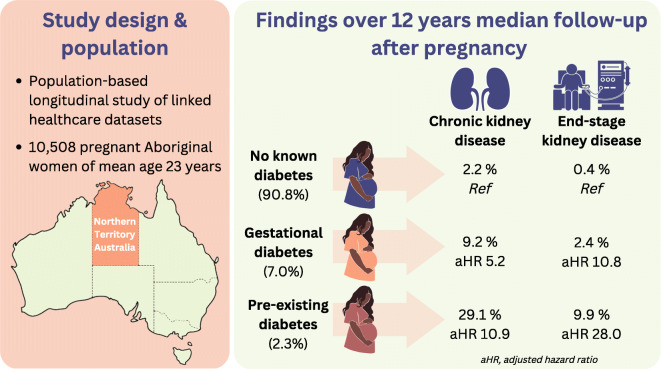

**Supplementary Information:**

The online version contains peer-reviewed but unedited supplementary material available at 10.1007/s00125-023-05868-w.

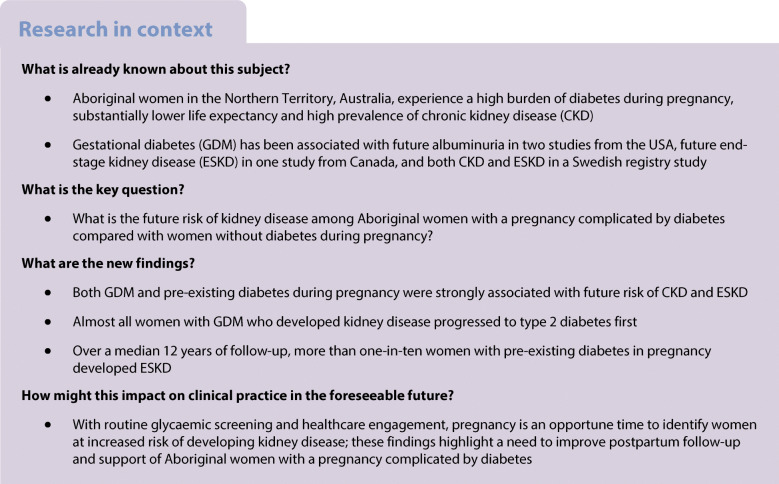



## Introduction

Indigenous populations impacted by colonisation are disproportionately affected by diabetes and related cardiometabolic conditions [1]. The prevalence of type 2 diabetes in Aboriginal people in the Northern Territory (NT), Australia, is among the highest of any population globally [2]. Onset is occurring at increasingly young ages, especially in women [3]. Rates of both gestational diabetes mellitus (GDM) and pre-existing type 2 diabetes in pregnancy are growing rapidly [4]. Of concern, Aboriginal women in the NT have 12.8 years shorter life expectancy than non-Aboriginal women [5], with diabetes being the most commonly attributed cause of death [6].

The burden of chronic kidney disease (CKD) for Aboriginal people in the NT is among the highest reported [7]; one-third of adults display biochemical evidence of CKD [8]. Furthermore, the age-standardised prevalence of end-stage kidney disease (ESKD) is estimated to be 1.8% for Aboriginal people compared with 0.1% for non-Aboriginal people [9]. Diabetes is a key contributing factor but there are multiple determinants with differing impacts across the life course [7, 10]. There is evidence that intergenerational risk factors, such as maternal nutrition and smoking, affect the intrauterine environment, leading to a reduction in nephron number from birth and greater susceptibility to CKD [11].

The prevalence of CKD is highest in remote and socioeconomically disadvantaged areas, where provision of renal replacement therapy is immensely challenging [7]. Aboriginal women in the NT have higher prevalence and incidence of ESKD than men [9]. The determinants of this difference are not known. Women are also less likely to access renal replacement therapy [9], possibly relating to reluctance to relocate hundreds of kilometres away from home due to family and community responsibilities [12].

Research into sex-specific risk factors for diabetes and kidney disease is warranted. Hypertensive disorders of pregnancy are known to predict future CKD and ESKD [13]. However, few studies have investigated the risk of subsequent CKD following a pregnancy complicated by diabetes [13–17]. Given near universal healthcare engagement and routine diabetes screening, pregnancy and the postpartum period present valuable opportunities for targeted prevention strategies.

The NT spans 1.35 million km^2^ (more than twice the size of France) but has a population density of just 0.2 people/km^2^ [18]. Almost one-third of the population identify as Aboriginal people and most live in remote communities [18]. Aboriginal people have lived in this region for more than 60,000 years. Traditional diet and lifestyle of many people have changed but great strength of culture persists, with more than 100 languages and dialects spoken [19].

In this context, we investigated risk of developing kidney disease following a pregnancy complicated by GDM or pre-existing diabetes among Aboriginal women.

## Methods

### Study design and population

**Fig. 1:**
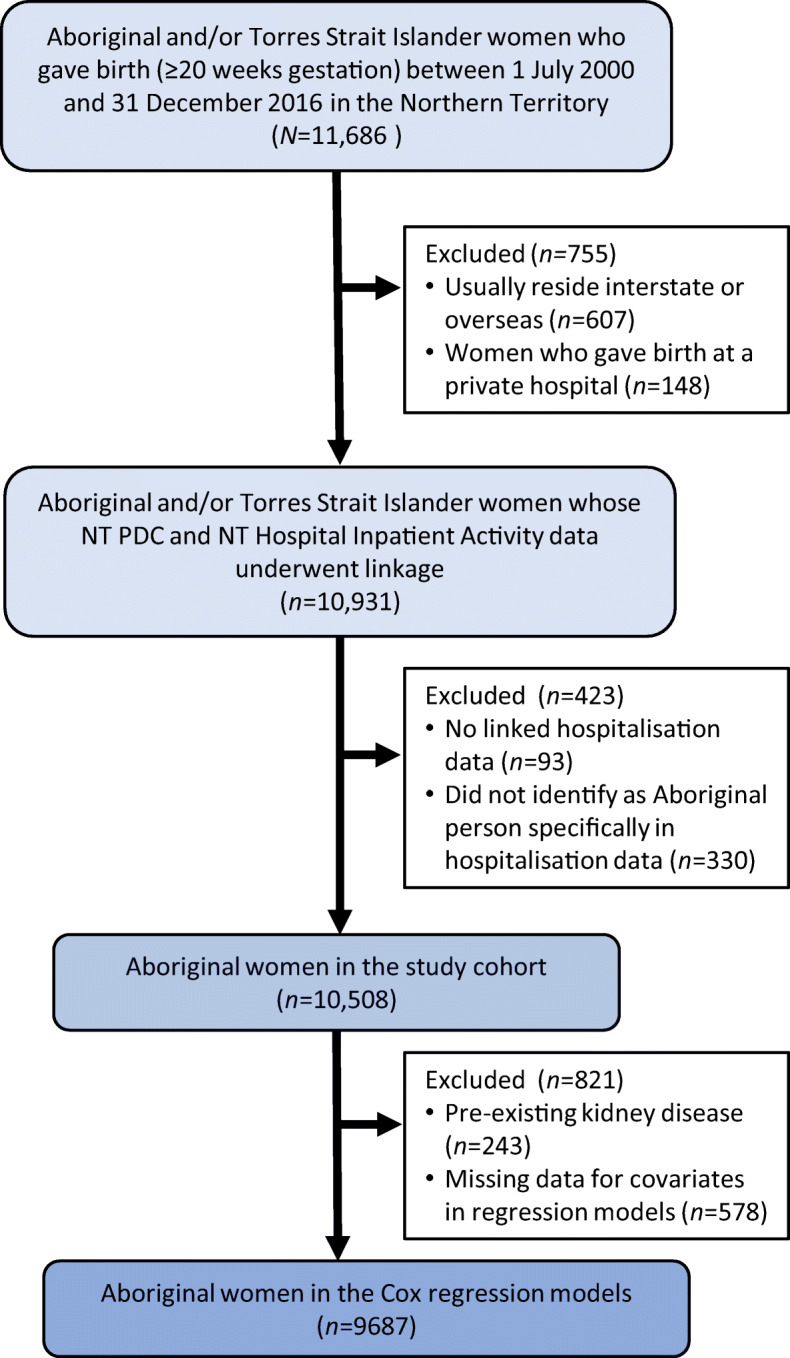
Study population flow chart showing inclusion and exclusion criteria

A longitudinal, population-based study was undertaken using linked healthcare datasets. All women, between 1 July 2000 and 31 December 2016, who identified as an Aboriginal person, gave birth at ≥20 weeks gestation and usually resided in the NT were eligible for inclusion. Women who gave birth at the one private hospital in the region were excluded as data could not be linked. Figure 1 summarises the cohort formation. For women with more than one pregnancy during the study period, only the earliest (‘index’) pregnancy was included.

Baseline data were extracted from the NT Perinatal Data Collection (NT PDC), a population-based register of all births, including non-hospital births. Investigators had access to the full database population and applied the inclusion and exclusion criteria to form the study population. The NT PDC contains detailed information about demographics, maternal health, antenatal care and perinatal outcomes. Follow-up outcome data between 1 July 2000 and 30 June 2018 were from the NT Hospital Inpatient Activity database. This provided ICD-10 (http://apps.who.int/classifications/icd10/browse/2016/en), Australian Modification (ICD-10-AM) codes for principal and secondary diagnoses for all admissions at every public (government-funded) hospital in the NT. Universal free access to hospital care is available in Australia. Loss to follow-up could not be identified in the study data. Nevertheless, the out-migration rate for Aboriginal people in the NT is low, with only 6% of the population moving away over a 5 year period [20].

Individual-level records were deterministically linked using a de-identified linkage key derived from each individual’s unique hospital reference number. These reference numbers have been used across all public health services in the NT since the early 1990s and have been reliably used in previous data-linkage studies [9]. In this study, there was 100% concordance in the sex of participants between the datasets and 99.0% of dates of birth matched to the day, indicating highly accurate linkage.

### Diabetes in pregnancy

Diabetes status during the index pregnancy was as recorded in the NT PDC, including GDM and pre-existing diabetes (type 1, type 2, or other diabetes). Data on the type of pre-existing diabetes was only available for 2014–2016. Women without recorded GDM or pre-existing diabetes were presumed to have no diabetes in the index pregnancy. This clinical information was entered by referring clinicians, usually midwives, immediately after a birth and thus represents the clinically known diabetes status during the pregnancy. To ensure capture of known diabetes status, the NT PDC has additionally been centrally cross-referenced against hospital files from the birth admission since 2008 and, from 2014, reporting of diabetes has been strengthened by data from the NT Diabetes in Pregnancy Clinical Register [21]. Some cases of diabetes will have been undiagnosed due to incomplete uptake of screening. Universal screening for GDM was recommended throughout the study period but screening approaches and diagnostic criteria did change (electronic supplementary material [ESM] Table 1). Glucose thresholds for the diagnosis of type 2 diabetes were unchanged over the study period, but HbA_1c_, at a threshold of ≥48 mmol/mol (≥6.5%), was adopted as a diagnostic option from 2012 [22].

### Ethnicity

First Nations peoples in Australia include both Aboriginal and Torres Strait Islander peoples. In the NT, 96% of the First Nations population identify as Aboriginal people [23]. To avoid misinterpretation regarding the generalisability of this study, the cohort was restricted to Aboriginal women specifically. Data from the NT PDC did not differentiate between Aboriginal and/or Torres Strait Islander identification. This more detailed information was available in the hospital data. Therefore, following data linkage, women identifying as Torres Strait Islander or both Aboriginal and Torres Strait Islander people were excluded.

### Other baseline variable definitions

Age was calculated at the day women gave birth. Usual location of residence at the time of the index pregnancy was recorded as free text in the NT PDC. The 438 different place names were manually designated into statistical areas of the Australian Statistical Geography Standard (ASGS), including Statistical Areas Level 1 (SA1s) and Indigenous Areas (IAREs) [24]. Using each individual’s SA1 code, remoteness was classified according to standard remoteness areas using the Accessibility/Remoteness Index of Australia (ARIA+), which is based on road distance from a location to the nearest urban centre [25]. Three out of five remoteness levels exist in the NT: outer regional; remote; and very remote. Area-level socioeconomic status was classified using the Indigenous Relative Socioeconomic Outcomes (IRSEO) index according to IAREs [26]. The IRSEO index is calculated using data from the 2016 Australian Census relating to the usual resident Aboriginal and/or Torres Strait Islander population of an area. It draws upon nine variables, covering employment, education, income and housing, to create a percentile score of relative socioeconomic outcomes. Scores range from 1 (most advantaged) to 100 (most disadvantaged). The region variable classified women as being from the ‘Top End’ or ‘Central Australia’. The Aboriginal population of the NT encompasses numerous distinct people groups. These regions are known to have marked differences in health outcomes [2]. Smoking status during the index pregnancy was missing for 1430 (13.6%) women. Many of these women had a subsequent pregnancy within the study period. For each woman missing smoking status in the index pregnancy, smoking status was assumed to be the same as in her most recent pregnancy. Following this imputation, smoking data were missing for 434 (4.1%) women. History of kidney disease pre-dating the index pregnancy was as recorded in the NT PDC or if a woman met the study’s outcome definitions in the linked hospital data prior to the index pregnancy. History of GDM in a previous pregnancy (previous GDM) was as recorded by clinicians in the NT PDC during the index pregnancy. Hypertensive disorders of pregnancy were as recorded in the NT PDC and included hypertension, pre-eclampsia and eclampsia arising in pregnancy or labour.

### Outcome definitions

Outcomes were captured from hospital admission coding. The two key outcomes were any CKD and ESKD. Any CKD incorporated ICD-10-AM codes for any stage of CKD or dialysis. ESKD included coding for stage 5 CKD or dialysis. Rates of type 2 diabetes among women with a history of GDM, who developed CKD or ESKD, were also explored. Analyses relating to incident type 2 diabetes were exploratory in nature only due to limited capture of events when relying on hospitalisation data. Most diagnoses of type 2 diabetes are made in the outpatient, primary care setting. For details of ICD-10-AM codes, see ESM Table 2. Date of death was obtained from hospital discharge data. Out-of-hospital deaths were not captured.

### Statistical analysis

Baseline descriptive data are presented as *n* (%), mean ± SD or median (IQR) as appropriate. Comparisons across categories of diabetes status during the index pregnancy were made using χ^2^ tests, one-way ANOVA and the non-parametric equality of medians test. The numbers of outcome events and crude rates (%) are presented by categories of diabetes status.

Kaplan–Meier estimates were used to calculate cumulative incidence of the pre-specified outcomes with 95% CIs. The risk of developing each outcome was compared using Cox proportional hazards models. Diabetes status in the index pregnancy was included as a three-level categorical variable, with both exposure categories assessed in the same regression models and compared against no known diabetes. Time origin was from the baby’s date of birth. Women with pre-existing kidney disease (*n*=243) were excluded. Subsequently, women missing data for smoking (*n*=419) and/or socioeconomic status (*n*=159) were also excluded. The date of the first event for each outcome was taken as the date of admission to hospital for the earliest admission in which a relevant ICD-10-AM code was recorded. Women were censored at the end of the follow-up period (30 June 2018) or on the date of death. The assumption of proportional hazards was verified by plotting the log of negative log of the survival function against the log of time and also by plotting the observed Kaplan–Meier survival curves against the Cox predicted curves. Crude and adjusted HRs were calculated. Variables included in the adjusted Cox models were chosen based on the plausibility of a clinically relevant relationship and assessment of associations with CKD in univariable or minimally adjusted models. The final adjusted model for the primary analysis included age, hypertensive disorder of pregnancy, smoking status, region and socioeconomic status (IRSEO score as a continuous variable). Available but excluded variables were parity, which had no association with CKD after accounting for maternal age, and multiple pregnancy (twin or triplet) and alcohol intake in pregnancy, neither of which had an association with CKD in univariable or multivariable models. Separate Cox models were used to examine associations between each secondary exposure and CKD and ESKD, with inclusion of potential confounders specific to each exposure [27].

Due to existing evidence showing that the relative risk of end-organ complications associated with having diabetes lessens with age [28, 29], it was pre-specified to look for an interaction between diabetes status and age using an interaction term in Cox models for the two key outcomes. Sensitivity analyses were undertaken to investigate any potential impact of excluding women with missing smoking data. Two opposite, extreme scenarios were applied to the missing data. First, it was assumed that women with diabetes smoked during pregnancy and women without diabetes did not smoke. Second, it was assumed that women with diabetes did not smoke during pregnancy and women without diabetes did smoke.

All analyses were conducted in Stata (V17.0; StataCorp, USA).

### Ethics and governance

The study was approved by the Human Research Ethics Committee of NT Health and Menzies School of Health Research, including the Aboriginal Ethics Sub-Committee (Ref: 2018-3069), with reciprocal approval from the Central Australian Human Research Ethics Committee (Ref: CA-19-3412). In addition, the Aboriginal and Torres Strait Islander Advisory Group of the Diabetes across the Lifecourse: Northern Australia Partnership supported the planned analyses and provided feedback on results. Authors VW and DH represent the Advisory Group in this publication. The Advisory Group will play an ongoing role in guiding knowledge translation.

## Results

In total, 10,508 Aboriginal women were included in the study (see Fig. 1). Mean age was 23.1 (SD 6.1) years, 731 (7.0%) women had GDM, 239 (2.3%) had pre-existing diabetes in pregnancy and 3451 (32.8%) were from Central Australia (rather than the Top End). Women with GDM or pre-existing diabetes were older, less likely to smoke and more likely to have a hypertensive disorder of pregnancy (Table 1). Pre-eclampsia accounted for most of the hypertensive disorders (*n*=750, 72.9%). Pre-existing kidney disease in the index pregnancy was more common among women with pre-existing diabetes (*n*/*N*=36/239, 15.1%) compared with women with GDM (*n*/*N*=13/731, 1.8%) or no known diabetes (*n*/*N*=194/9538, 2.0%). After a median (IQR) follow-up time of 12.1 (7.2–15.8) years, 334 (3.3%) women developed CKD, 70 (0.7%) developed ESKD and 54 (0.5%) died.

**Table 1:** Characteristics of the study cohort stratified by diabetes status during the index pregnancy

Characteristic	*N*	No known diabetes (*n*=9538)	Gestational diabetes (*n*=731)	Pre-existing diabetes (*n*=239)	*p* value
Baseline characteristics					
Age, years	10,508	22.6 ± 5.8	26.6 ± 6.7	31.5 ± 6.9	<0.001
Central Australia region	10,508	3101 (32.5)	238 (32.6)	112 (46.9)	<0.001
Remoteness	10,445				0.380
Outer regional		1384 (14.6)	94 (13.0)	27 (11.3)	
Remote		2046 (21.6)	163 (22.5)	59 (24.7)	
Very remote		6051 (63.8)	468 (64.6)	153 (64.0)	
Socioeconomic index (IRSEO)	10,338	90.1 (77.0–95.3)	90.4 (77.0–95.3)	91.9 (79.2–96.1)	0.110
Smoking during pregnancy	10,074	4416 (48.2)	320 (46.3)	87 (40.1)	0.043
Pre-existing kidney disease	10,508	194 (2.0)	13 (1.8)	36 (15.1)	<0.001
Previous GDM	10,508	29 (0.3)	165 (22.6)	24 (10.0)	<0.001
Hypertensive disorder of pregnancy	10,508	880 (9.2)	101 (13.8)	48 (20.1)	<0.001
Nulliparity	10,508	6129 (64.3)	402 (55.0)	78 (32.6)	<0.001
Follow-up information					
Follow-up, years	10,508	12.3 (7.4–15.9)	10.4 (5.0–14.4)	11.2 (6.5–16.1)	<0.001
No. of hospitalisations	10,508	4 (2–6)	4 (2–8)	8 (4–16)	<0.001

Data are shown as *n* (%), mean ± SD or median (IQR)

**Fig. 2:**
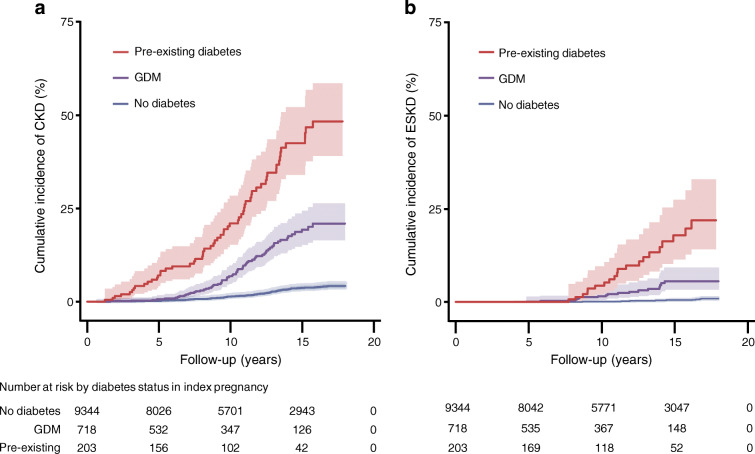
Cumulative incidence (95% CI) of any CKD (**a**) and ESKD (**b**) according to diabetes status during the index pregnancy of Aboriginal women in the NT, Australia

Compared with women without diabetes during their index pregnancy, the incidence of any CKD and ESKD during follow-up was higher for women with GDM and highest for women with pre-existing diabetes in pregnancy (Fig. 2, Table 2). After adjustment for potential confounding factors, there was strong evidence for increased risk of any CKD among women with GDM (HR 5.2 [95% CI 3.9, 7.1]) and women with pre-existing diabetes in pregnancy (HR 10.9 [95% CI 7.7, 15.5]) compared with women without diabetes in pregnancy. An even greater elevation in risk was seen for ESKD with both GDM (HR 10.8 [95% CI 5.6, 20.8]) and pre-existing diabetes in pregnancy (HR 28.0 [95% CI 13.4, 58.6]). Among women with GDM, 62 (93.9%) of those who developed any CKD and 16 (94.1%) of those who developed ESKD were known to have progressed to type 2 diabetes prior to onset of kidney disease. In 2014–2016, when data on pre-existing diabetes type were available, all 36 women with pre-existing diabetes in pregnancy had type 2 diabetes.

**Table 2:** Absolute and relative risk of future kidney disease according to diabetes status during index pregnancy among Aboriginal women in NT, Australia

Kidney disease	Event rates, *n* (%)			Crude HR vs no diabetes during index pregnancy (95% CI)		Adjusted HR vs no diabetes during index pregnancy (95% CI)^a^	
	No known diabetes (*n*=9344)	GDM (*n*=718)	Pre-existing diabetes (*n*=203)	GDM	Pre-existing diabetes	GDM	Pre-existing diabetes
Any CKD	209 (2.2)	66 (9.2)	59 (29.1)	6.1 (4.5, 8.1)	17.2 (12.6, 23.4)	5.2 (3.9, 7.1)	10.9 (7.7, 15.4)
ESKD	33 (0.4)	17 (2.4)	20 (9.9)	11.2 (6.0, 20.8)	35.7 (19.2, 66.7)	10.8 (5.6, 20.8)	28.0 (13.4, 58.6)

Women missing data for smoking (*n*=419) and socioeconomic status (*n*=159) were excluded from both the crude and adjusted Cox models (*n*=9687 included)

^a^Adjusted for maternal age, hypertensive disorder of pregnancy, socioeconomic status, region and smoking status during pregnancy

In separate Cox regression analyses designed to assess the effects of secondary exposure variables, hypertensive disorders of pregnancy, maternal age, socioeconomic disadvantage (IRSEO score), residing in a very remote location and being from the Central Australia region were associated with increased risk of CKD (ESM Table 3). Maternal age, socioeconomic disadvantage and being from Central Australia were also associated with ESKD (ESM Table 4).

Clinical and demographic characteristics of women excluded due to missing data vs those included in the Cox regression models are presented in ESM Table 5. Excluded women were older, more likely to have diabetes, and more likely to live in a very remote area. Nevertheless, sensitivity analyses suggested no impact of their exclusion on the associations of GDM and pre-existing diabetes with future CKD or ESKD (ESM Table 6).

The relative risk inferred by having a history of pre-existing diabetes in pregnancy lessened with age for both CKD (*p*=0.004 for interaction) and ESKD (*p*<0.001 for interaction). Event rates and HRs among women with pre-existing diabetes in pregnancy, stratified by age, are presented in ESM Table 7. There were no significant interactions between age and GDM status.

## Discussion

In this relatively young cohort of parous Aboriginal women with a long duration of follow-up, there was strong evidence that both GDM and pre-existing diabetes (predominantly type 2 diabetes) in pregnancy are associated with high risk of future CKD and ESKD. Other risk factors identified for future CKD included age, hypertensive disorders of pregnancy, living in remote areas, socioeconomic disadvantage and being from the Central Australia region. While the relative risk of kidney disease compared with women without diabetes during pregnancy was remarkably high, the absolute event rates are equally concerning, with almost one in ten women with GDM developing CKD and a similar proportion of women with pre-existing diabetes developing ESKD.

This is the first study to investigate long-term kidney disease outcomes after diabetes in pregnancy among Aboriginal women in Australia. The findings suggest potential modifiable risk factors for kidney disease that are specific to parous women. Compared with Aboriginal men, women have a higher prevalence of ESKD (2.1% in women vs 1.5% in men) and an accelerated decline in eGFR over time has been observed [9, 10]. This disparity may in part relate to dysglycaemia, which can be detected relatively early in the life course, during or prior to pregnancy.

In this study, almost all of the women with GDM, who later developed kidney disease, progressed to having type 2 diabetes prior to kidney disease onset. Therefore, the risk associated with GDM was not shown to be independent of future type 2 diabetes. Nevertheless, GDM provides an early risk marker that could potentially enable targeted screening and prevention activities. A previous data-linkage study from Ontario demonstrated increased risk of ESKD among women with GDM who subsequently developed type 2 diabetes [16]. Similar to our study, the relative risk in that cohort, compared with women with no history of GDM and no subsequent type 2 diabetes, was very high (HR 7.5 [95% CI 5.2, 10.8]). Another study examined associations between GDM and future CKD and ESKD in Swedish national registry data [17]. The relative risks among women with GDM and subsequent type 2 diabetes, compared with women without GDM, were remarkably high for CKD (adjusted HR 21.7 [95% CI 17.2, 27.4]) and ESKD (adjusted HR 112.4 [95% CI 61.2, 206.4]). In keeping with our findings, women with GDM who did not progress to type 2 diabetes were not at increased risk of kidney disease [17].

These Canadian and Swedish studies were not included in a 2020 systematic review, which did not find any studies showing an association between GDM and future ESKD [13]. With regard to earlier stages of CKD, a prospective study from the USA found that GDM was predictive of future albuminuria over 21 years of follow-up among Black women but not White women [15]. Similarly, in a cross-sectional analysis from the Kidney Early Evaluation Program (KEEP) in the USA, self-reported history of GDM without subsequent type 2 diabetes was associated with risk of microalbuminuria but not later stages of CKD [14]. When stratified by ethnicity, evidence for this association was present among Black participants but not White participants. The existence of similarities between minority groups in the USA and our study population likely reflect shared risks relating to socioeconomic disadvantage. The stronger associations with kidney disease observed in our study are presumably driven by higher rates of progression to chronic impaired glucose regulation among Aboriginal women in the NT. A prospective cohort study in the NT showed that within just 2.5 years’ follow-up postpartum, 22% of Aboriginal women with GDM had developed type 2 diabetes and an additional 11% had intermediate hyperglycaemia (HbA_1c_ ≥6.0%, impaired glucose tolerance or impaired fasting glycaemia) [30].

The high incidence of CKD and ESKD in this relatively young cohort with pre-existing diabetes during pregnancy is consistent with studies of young-onset type 2 diabetes [31]. The TODAY (Treatment Options for Type 2 Diabetes in Adolescents and Youth) trial enrolled participants with type 2 diabetes aged 10–17 years [32]. Over 15 years, the cumulative incidence of moderate or severe albuminuria was 54.7%. A relatively high incidence of kidney disease in individuals with young-onset type 2 diabetes has been observed in other Indigenous populations, including Akimel O’odham (Pima) people from Arizona, USA and First Nations people from Saskatchewan and Manitoba, Canada [33–35].

The additional risk factors associated with CKD in our study are consistent with existing evidence but should be interpreted with caution given that diabetes status in pregnancy was the primary exposure of interest. Our findings emphasise the importance of considering the social determinants of health and equity of access to culturally appropriate health services.

Strengths of our study include its whole-of-population approach, large sample size and duration of follow-up in a high-priority, marginalised population group. The accurate data-linkage methodology facilitated valuable research in a context where traditional epidemiological studies are difficult to undertake for numerous reasons, including the vast distances between many small population centres. Additionally, we were able to look at robust clinical outcomes rather than just surrogate markers of risk. Importantly, the study is embedded in a larger programme of work with strong Aboriginal representation and governance processes as well as meaningful collaborative partnerships that help facilitate translation of research findings into policy and practice [36].

The study has several limitations, largely relating to the retrospective approach and use of existing datasets. The classification of diabetes status during pregnancy was limited to known diagnoses and assessed categorically without any glycaemic measures available. There were changes in the screening and diagnosis of GDM during the study period. However, the proportionality of the HRs over time suggests that the risk inferred by a diabetes diagnosis at different time points across the study period was consistent. While there is universal access to free healthcare in Australia, uptake of GDM screening will not have been complete. There will have been some women with undiagnosed diabetes in the reference group, contributing to likely underestimation of risks associated with both GDM and pre-existing diabetes in pregnancy. Recording of kidney disease that pre-dated the index pregnancy, especially early CKD, may also have been incomplete. Our findings do not show that having a pregnancy complicated by diabetes is causative of heightened CKD risk, but rather that diabetes detected either during or prior to pregnancy strongly predicts future development of CKD. The same outcomes could potentially be seen among nulliparous women with young-onset diabetes.

The use of hospital coding data for outcome capture is highly reliable for ESKD, as dialysis initiation services are provided by the public hospital system and hospitalisation rates are high in people with ESKD irrespective of dialysis use. However, detection of early stages of CKD and future type 2 diabetes will not have been complete. Early CKD and type 2 diabetes are diagnoses predominantly made in the primary care setting, so capture from hospital data is largely dependent on women being admitted for another reason. The diagnosis of CKD is more likely to have been documented for later stages of CKD due to the clinical relevance to an acute admission. There is also potential for detection bias with regard to CKD and type 2 diabetes because the risk of hospitalisation may vary between the diabetes in pregnancy exposure groups. However, the median number of hospitalisations during the study period was the same for women with GDM and women with no diabetes in pregnancy. The median number of hospitalisations was greater for women who had pre-existing diabetes during the index pregnancy, which could have increased detection of CKD diagnoses among this group. Nevertheless, the consistency of the findings relating to CKD and ESKD (ESKD being reliably detected in hospital data) suggest that the associations observed with CKD are true. Mortality data were also reliant on hospital records. It is possible that some women may have died without being hospitalised. Given the young age of the cohort, it is unlikely that capturing out-of-hospital deaths would alter the findings.

Another limitation is the potential for unmeasured confounding in our analyses, in particular from obesity but also other known risk factors such as recurrent infections and family history of kidney disease. Existing evidence suggests that cumulative exposure to GDM in more than one pregnancy is associated with greater risk of CKD [37]. Due to the design of our linked study database, we were unable to reliably investigate this hypothesis. Finally, our findings are only generalisable to the Aboriginal population of the NT but may be of relevance to the broader Aboriginal population of Australia and other Indigenous populations globally with high rates of diabetes and kidney disease.

In conclusion, GDM and pre-existing diabetes during pregnancy are strongly associated with increased risk of developing CKD and ESKD among Aboriginal women in the NT of Australia. Pregnancy presents an important opportunity to identify kidney disease risk. Our findings also highlight the importance of ongoing screening for type 2 diabetes after a pregnancy complicated by GDM. Culturally appropriate strategies to prevent kidney disease in this high-risk population should be investigated alongside public health strategies that address the social determinants of health and improve access to healthcare services.

## Supplementary information

ESM (PDF 705 kb)

## Data Availability

NT Health, NT Government, is the owner of the datasets used in this study. Any access to the deidentified study dataset requires relevant governance approvals from NT Health. Information regarding accessing NT Health data can be found at https://health.nt.gov.au/data-and-research/health-data/how-to-request-data-for-secondary-use.

## Abbreviations

CKD: Chronic kidney disease
ESKD: End-stage kidney disease
GDM: Gestational diabetes mellitus
IARE: Indigenous Area
ICD-10-AM: ICD-10, Australian Modification
IRSEO: Indigenous Relative Socioeconomic Outcomes
NT: Northern Territory
NT PDC: NT Perinatal Data Collection
SA1: Statistical Areas Level 1
