# Supplementary material for: Risk of kidney disease following a pregnancy complicated by diabetes: a longitudinal, population-based data-linkage study among Aboriginal women in the Northern Territory, Australia
Source: Diabetologia. 2023 Jan 18;66(5):837–46. doi: 10.1007/s00125-023-05868-w (PMC10036460; doi:10.1007/s00125-023-05868-w)
Supplement: Supplementary file 1 — (PDF 705 kb) [file 125_2023_5868_MOESM1_ESM.pdf]

ESM Table 1. Relevant guidelines for the screening and diagnosis of gestational diabetes during the study period [1-3]

Time period	Organisation(s)	Screening approach	Diagnostic test	Thresholds
2000-2013[a]	ADIPS (modified from WHO)	Initial non-fasting 50g glucose challenge (threshold 7·8 mmol/L). Universal.	2-hour 75g OGTT	FPG ≥5·5 mmol/L<br>2hPG ≥8·0 mmol/L
2013 onwards[b]	ADIPS (in line with IADPSG and WHO)	1-step. Universal.	2-hour 75g OGTT	FPG ≥5·1 mmol/L<br>1hPG ≥10·0 mmol/L<br>2hPG ≥8·5 mmol/L

(a) These national guidelines were in place from 1991. (b) Gradually implemented into local Northern Territory policies across 2014-2015. OGTT = oral glucose tolerance test, ADIPS = Australasian Diabetes in Pregnancy Society, WHO = World Health Organization, FPG = fasting plasma glucose, 1hPG = 1-hour plasma glucose, 2hPG = 2-hour plasma glucose, IADPSG = International Association of Diabetes in Pregnancy Study Groups.

References:

1. Hunter A, Doery JC, Miranda V. Diagnosis of gestational diabetes in Australia: a national survey of current practice. Med J Aust 1990; 153(5):290-2.

2. Martin FI. The diagnosis of gestational diabetes. Ad Hoc Working Party. Med J Aust 1991; 155(2):112.

3. Australasian Diabetes in Pregnancy Society (2013). ADIPS Consensus Guidelines for the Testing and Diagnosis of Gestational Diabetes Mellitus in Australia.

ESM Table 2. International Classification of Diseases – 10th Revision – Australian Modification (ICD-10-AM) codes for outcome variables

Outcome	**Description** (years codes were in use)	ICD-10-AM codes
Any CKD	Stages 1-5 CKD (2009-2018)	N18.1, N18.2, N18.3, N18.4, N18.5
	End stage renal disease (2000-2008)	N18.0
	Other chronic renal failure (2000-2008)	N18.8
	Unspecified CKD (2000-2018)	N18.9*
	Dependence on renal dialysis (2000-2018)	Z99.2
	Preparatory care for dialysis (2000-2018)	Z49.0
	Extracorporeal dialysis (2000-2018)	Z49.1
ESKD	Stage 5 CKD (2009-2018)	N18.5
	End stage renal disease (2000-2008)	N18.0
	Dependence on renal dialysis (2000-2018)	Z99.2
	Preparatory care for dialysis (2000-2018)	Z49.0
	Extracorporeal dialysis (2000-2018)	Z49.1
Type 2 diabetes	Type 2 diabetes mellitus	E11*
	Diabetes mellitus in pregnancy: Pre-existing type 2 diabetes mellitus	O24.1*

CKD, chronic kidney disease; ESKD, end stage kidney disease. *All codes beginning with.

ESM Table 3. Effect estimates for associations between secondary exposure variables and chronic kidney disease (CKD) calculated using separate Cox proportional hazards models.

Secondary Exposure Variable	Crude Hazard Ratio (95% CI) (n=9687)	Adjusted Hazard Ratio (95% CI) (n=9687)
Age, 5-year increments	1.4 (1.3-1.5)	1.5 (1.2-1.6)[a]
Remoteness		
Outer Regional	*Ref*	*Ref*
Remote	2.8 (1.4-5.3)	1.8 (0.9-3.6)[b]
Very Remote	4.6 (2.5-8.4)	4.0 (2.2-7.4)[b]
Socioeconomic disadvantage (per ISREO decile)	1.2 (1.1-1.3)	1.2 (1.2-1.3)[c]
Hypertensive disorder of pregnancy	3.0 (2.3-4.0)	2.5 (1.9-3.3)[d]
Smoking in index pregnancy	0.7 (0.6-0.9)	0.8 (0.7-1.1)[e]
Central Australia region (vs Top End)	1.8 (1.5-2.3)	1.7 (1.3-2.1)[f]

IRSEO, Indigenous Relative Socioeconomic Outcomes (higher score = greater disadvantage). Models adjusted for: (a) IRSEO, smoking status and region; (b) smoking status, region, diabetes status and hypertensive disorders of pregnancy; (c) region, age, diabetes status, hypertensive disorder of pregnancy and smoking status; (d) age, diabetes status, IRSEO, smoking status and region; (e) age, diabetes status, IRSEO and region; (f) age, diabetes status, hypertensive disorder of pregnancy, IRSEO and smoking status.

ESM Table 4. Effect estimates for associations between secondary exposure variables and end stage kidney disease (ESKD) calculated using separate Cox proportional hazards models.

Secondary Exposure Variable	Crude Hazard Ratio (95% CI) (n=9687)	Adjusted Hazard Ratio (95% CI) (n=9687)
Age, 5-year increments	1.5 (1.2-1.8)	1.5 (1.2-1.8)[a]
Remoteness		
Outer Regional	*Ref*	*Ref*
Remote	2.8 (0.8-9.7)	1.5 (0.4-5.5)[b]
Very Remote	2.7 (0.8-8.6)	1.9 (0.6-6.4)[b]
Socioeconomic disadvantage (per ISREO decile)	1.1 (1.0-1.2)	1.1 (1.0-1.2)[c]
Hypertensive disorder of pregnancy	2.2 (1.1-4.3)	1.5 (0.8-3.0)[d]
Smoking in index pregnancy	0.6 (0.3-1.0)	0.7 (0.4-1.2)[e]
Central Australia region (vs Top End)	2.5 (1.5-4.1)	1.9 (1.1-3.3)[f]

IRSEO, Indigenous Relative Socioeconomic Outcomes (higher score = greater disadvantage). Models adjusted for: (a) IRSEO, smoking status and region; (b) smoking status, region, diabetes status and hypertensive disorders of pregnancy; (c) region, age, diabetes status, hypertensive disorder of pregnancy and smoking status; (d) age, diabetes status, IRSEO, smoking status and region; (e) age, diabetes status, IRSEO and region; (f) age, diabetes status, hypertensive disorder of pregnancy, IRSEO and smoking status.

ESM Table 5. Comparison of clinical and demographic characteristics of women included and excluded from the Cox hazards models due to missing data.

	**Included (n=9687)**	**Excluded (n=578)**	**P-value**
Baseline characteristics			
Diabetes status in index pregnancy			<0.001
GDM	662 (6.8)	56 (9.7)	
Pre-existing diabetes	180 (1.9)	23 (4.0)	
Age, years	23.0 ± 6.0	24.0 ± 7.0	<0.001
Remoteness			0.001
Outer Regional	1429 (14.8)	64 (12.4)	
Remote	2143 (22.1)	85 (16.5)	
Very Remote	6115 (63.1)	367 (71.1)	
Previous GDM	186 (1.9)	24 (4.2)	<0.001
Hypertensive disorder of pregnancy	906 (9.4)	65 (11.3)	0.131
Nulliparity	6118 (63.2)	356 (61.6)	0.449
Follow-up information			
Follow-up, years	12.1 (7.1-15.8)	11.6 (8.7-15.9)	0.124
No. of hospitalisations	4 (2-6)	3 (2-5)	<0.001

Data are n(%), mean ± SD, median (IQR). Comparisons between groups are made with chi-square tests, unpaired t-tests, and Wilcoxon rank-sum tests as appropriate. IRSEO, Indigenous Relative Socioeconomic Outcomes; GDM, gestational diabetes.

ESM Table 6. Sensitivity analysis exploring potential effect of missing smoking status in pregnancy data using best-case (B) and worst-case (C) scenarios compared to complete case analysis (A).

	**aHR for GDM** vs. no diabetes during index pregnancy (95% CI)			**aHR for pre-existing diabetes** vs. no diabetes during index pregnancy (95% CI)		
	A	B	C	A	B	C
CKD	5.2 (3.9-7.1)	4.8 (3.6-6.5)	4.9 (3.7-6.6)	10.9 (7.7-15.5)	10.6 (7.6-14.7)	10.8 (7.7-15.0)
ESKD	10.8 (5.6-20.8)	9.5 (5.1-17.7)	9.7 (5.2-18.2)	28.0 (13.4-58.6)	27.0 (13.7-53.2)	27.9 (14.2-55.0)

aHR, adjusted hazard ratio; CKD, chronic kidney disease; ESKD, end stage kidney disease; CI, confidence interval. Models adjusted for age, hypertensive disorder in pregnancy, socioeconomic status, region and smoking status during pregnancy. Women missing data for socioeconomic status (n=159) were excluded. In scenario A, women missing smoking status (after imputation described in methods) are excluded (n=419). In scenario B, women with the exposure (GDM or pre-existing diabetes) who are missing smoking data are assumed not to smoke while women without diabetes during pregnancy are assumed to smoke. In scenario C, the opposite is implemented (women with the exposure are all assumed to smoke while women without the exposure are assumed not to smoke).

ESM Table 7. Absolute and relative risk of future kidney disease among women with pre-existing diabetes compared to women with no diabetes in pregnancy, stratified by age (years).

	**Event rates, n(%)**				**Crude Hazard Ratio** vs. no diabetes during index pregnancy (95% CI)		**Adjusted Hazard Ratio** vs. no diabetes during index pregnancy (95% CI)[a]	
	**No known diabetes & age <25** (n=6,726)	**Pre-existing diabetes & age <25** (n=44)	**No known diabetes & age ≥25** (n=2,618)	**Pre-existing diabetes & age ≥25** (n=159)	**Age <25** (n=6740)	**Age ≥25** (n=2957)	**Age <25** (n=6740)	**Age ≥25** (n=2957)
**Any CKD**	116 (1.7)	27 (38.6)	93 (3.6)	42 (26.4)	34.4 (20.0-59.2)	10.8 (7.3-15.8)	29.5 (17.1-51.0)	7.6 (5.0-11.6)
**ESKD**	15 (0.2)	10 (22.7)	18 (0.7)	10 (6.3)	150.2 (60.9-370.8)	13.5 (5.6-32.2)	120.9 (48.0-304.6)	9.5 (3.7-24.3)

CKD, chronic kidney disease; ESKD, end stage kidney disease; CI, confidence interval. (a) Adjusted for age, hypertensive disorder in pregnancy, socioeconomic status, region and smoking status during pregnancy. Women missing data for smoking (n=419) and socioeconomic status (n=159) were excluded from both the crude and adjusted Cox models.
